# Dentistry in Germany

**Published:** 1887-08

**Authors:** 


### Dentistry in Germany.

There are at present 1,435 people engaged in dentistry in the German Empire. Of this number, 505 are "approved" in Germany, 33 of whom are American graduates. Of the 94 practitioners who are graduates of American colleges, only 17 are females; 21 are "approved" in other countries, but not in America. There are 815 persons practicing mechanical dentistry without any other occupation. In the province of Hesse-Nassau there are 83 practitioners of dentistry, of whom 29 are "approved" 4 being American graduates, and 14 from American schools exclusively, 3 of whom are females. Two of these are "approved" in other countries (not America), and 38 mechanical dentists without other occupatian. In 77 cities with over 10,000 inhabitants, there is not a dentist, surgical or mechanical, and in 86 cities there are no surgeons, though there are in these places practitioners of mechanical dentistry.—*Jour. f. Zahnheilkunde.*

